# Anti-proliferative role of recombinant lethal toxin of *Bacillus anthracis* on primary mammary ductal carcinoma cells revealing its therapeutic potential

**DOI:** 10.18632/oncotarget.16214

**Published:** 2017-03-15

**Authors:** Rekha Khandia, Bramhadev Pattnaik, Katherukamem Rajukumar, Atul Pateriya, Sandeep Bhatia, Harshad Murugkar, Anil Prakash, Hare Krishna Pradhan, Kuldeep Dhama, Ashok Munjal, Sunil K. Joshi

**Affiliations:** ^1^ ICAR-National Institute of High Security Animal Diseases, Bhopal, Madhya Pradesh, India; ^2^ Project Directorate on Foot and Mouth Disease, Mukteswar, Uttarakhand, India; ^3^ Department of Microbiology, Barkatullah University, Bhopal, Madhya Pradesh, India; ^4^ Ex-Avian Influenza National Consultant, Indian Office of WHO Consultant, Bhartiya Kala Kendra, New Delhi, India; ^5^ Division of Pathology, ICAR-Indian Veterinary Research Institute, Izatnagar, Bareilly Uttar Pradesh, India; ^6^ Department of Biochemistry and Genetics, Barkatullah University, Bhopal, Madhya Pradesh, India; ^7^ Cellular Immunology Laboratory, Frank Reidy Research Center of Bioelectrics, College of Health Sciences, Old Dominion University, Norfolk, VA USA

**Keywords:** anthrax toxin receptor, bacillus anthracis, c-Met receptor, lethal factor, protective antigen

## Abstract

*Bacillus anthracis* secretes three secretary proteins; lethal factor (LF), protective antigen (PA) and edema factor (EF). The LF has ability to check proliferation of mammary tumors, chiefly depending on mitogen activated protein kinase (MAPK) signaling pathway. Evaluation of therapeutic potential of recombinant LF (rLF), recombinant PA (rPA) and lethal toxin (rLF + rPA = LeTx) on the primary mammary ductal carcinoma cells revealed significant (*p* < 0.01) reduction in proliferation of tumor cells with mean inhibition indices of 28.0 ± 1.37% and 19.6 ± 1.47% respectively. However, treatment with rPA alone had no significant anti-proliferative effect as evident by low mean inhibition index of 3.4 ± 3.87%. The higher inhibition index observed for rLF alone as compared to LeTx is contrary to the existing knowledge on LF, which explains the requirement of PA dependent endocytosis for its enzymatic activity. Therefore, the plausible existence of PA independent mode of action of LF including direct receptor mediated endocytosis or modulation of signal transduction cascade via unknown means is hypothesized. *In silico* protein docking analysis of other cellular receptors for any plausibility to play the role of receptor for LF revealed c-Met receptor showing strongest affinity for LF (H bond = 19; Free energy = −773.96), followed by nerve growth factor receptor (NGFR) and human epidermal growth factor receptor (HER)-1. The study summarizes the use of rLF or LeTx as therapeutic molecule against primary mammary ductal carcinoma cells and also the c-Met as potential alternative receptor for LF to mediate and modulate PA independent signal transduction.

## INTRODUCTION

Cancer remains a deadly malady despite several scientific advances and is one of the leading causes of deaths and high sufferings to the mankind. Though conventional therapies including of radiotherapy, chemotherapy and surgery are being followed widely; however due to their some limitations and side effects, researchers are continuously in the search of novel and alternative/complementary therapeutic options for countering various kinds of cancers and tumorous conditions. Some of such therapeutic regimens being explored include hormones inhibitors, immunotherapy (adjuvants, cytokines, TLR-agonists, immune-checkpoint inhibitors), apoptins (selective anti-cancer viral proteins), cryotherapy, molecular therapy (gene therapy, RNAi, CRISPR, Phages), homing peptides, herbs and plant metabolites, nanotechnology-based drug delivery as well as tumor vaccines, DNAzymes, HSP90 chaperone complex inhibitors, probiotic therapy, ribosome inactivating plant toxins, zootoxins derived from bees, snakes or scorpion, sponge toxins like agelasine B, or bacterial toxins and many others [1–10]. Several bacterial toxins are manipulated to specifically target tumor cells. These toxins include *Clostridium difficile* toxin [11, 12] Shiga-like toxin 1 [13, 14], *Pseudomonas* exotoxin A (PE) [15], *Pertussis* toxin [16] etc. Likewise, the same has been observed with lethal toxin of *Bacillus anthracis* [17]. In this direction, the present study reports the therapeutic role of recombinant lethal toxin of *Bacillus anthracis*, an etiological agent of anthrax, on primary mammary ductal carcinoma cells.

**Figure 1:**
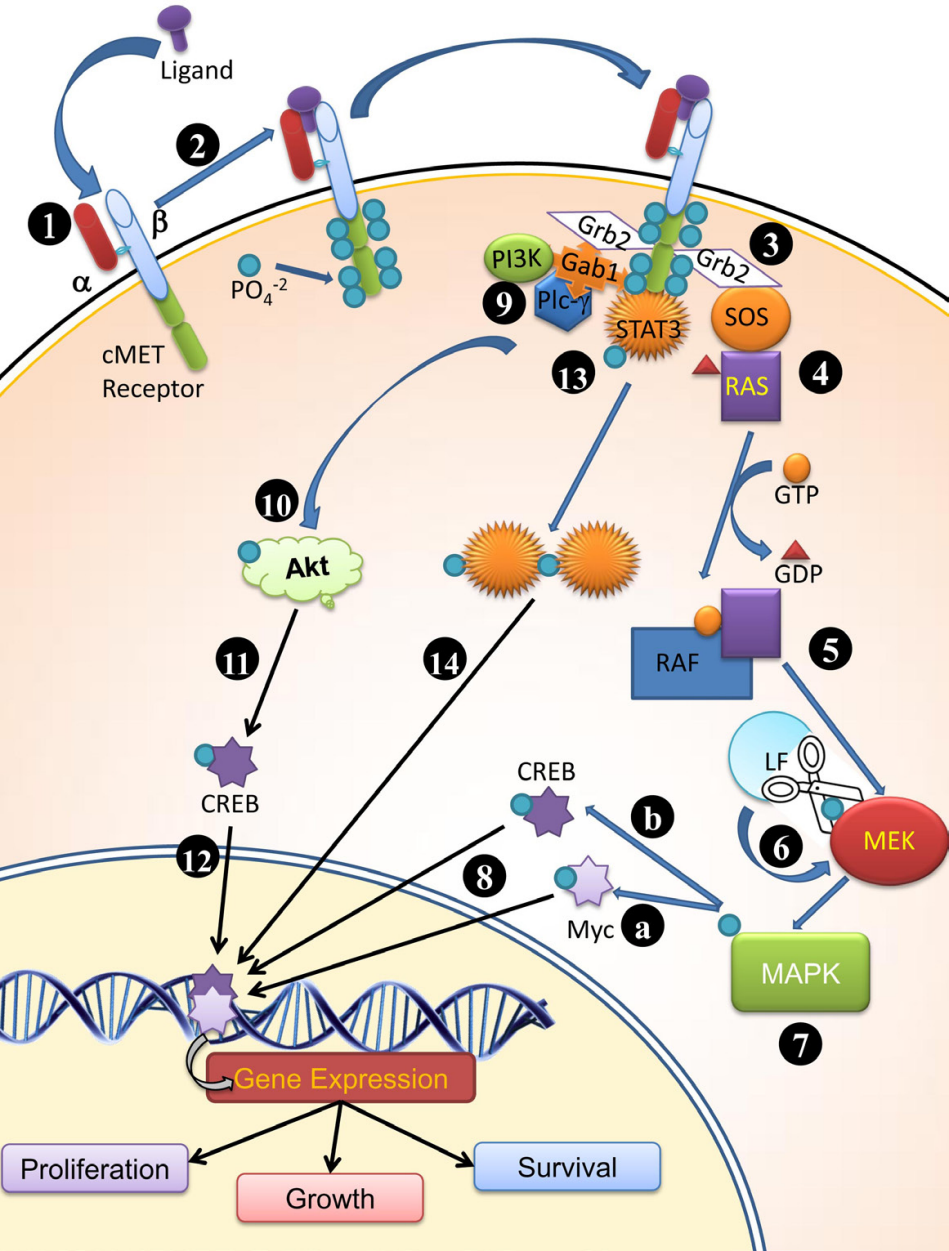
Plausible mode of functioning of cMET receptor (1) cMET is synthesized by hepatocytes. α subunit is extracellular; whereas the β subunit is trans-membrane peptide possessing a kinase domain and docking site for molecule which participate in cell signaling and receptor bioactivity (2) upon ligand binding to the cMET receptor, the tyrosine kinase domain is highly phosphorylated at tyrosine residue (1234–1235, 1349, 1356 at C terminus of β subunit) (3) Grb2 effecter binds to phosphorylated tyrosine kinase and RAS guanine exchange factor SOS (Son of sevenless) (4) SOS promotes dissociation of GDP from Ras and attachment of GTP thereby activates Ras (5) Ras activates Raf and in turn (6) Phosphorylates MEK, followed by phosphorylation of MAPK; LF cleaves MEKs and prevent further downstream signaling required for cell proliferation, survival and growth. (7) MAPK activates Myc (7A) and CREB (7B) by phosphorylation and (8) These translocates into nucleus and bind to their respective response elements (9) Gab1 interacts with cMet receptor and provide binding site for SH2 domain containing proteins (Grb2, PI3K, PLCγ) (10) PI3K phosphorylates Akt, which in turn (11) phosphorylates CREB and (12) allow transcription of surviving genes (also 7B) (13) Post phosphorylation C terminus of β subunit of the receptor acts as docking site for STAT3 and STAT3 is phosphorylated (14) Dimerized and translocated to nucleus for promoting different gene expressions.

*B. anthracis* contains two toxin-encoding plasmids, namely, pXO1 and pXO2. The 181 kb pXO1 encodes for lethal factor (LF), protective antigen (PA) and edema factor (EF). The pXO2 encodes for the bacterial capsule, which prevents its phagocytosis by host immune cells [18]. Proteolysis of the mature PA, also known as PA83, by furin like proteases present in host cells, yields a 20 kDa amino-terminal fragment, PA20 and a 63 kDa carboxyl-terminal fragment, PA63 [19]. The biologically active PA63 forms a heptamer of PA63 which facilitates the binding and entry of LF and EF into the host cell cytoplasm through receptor mediated endocytosis [20]. The combination of LF and PA is called Lethal Toxin (LeTx). Lethal factor is a zinc dependent metalloprotease of 89 kDa size and contains zinc-binding motif, HEXXH [21]. The substrates for LF are mitogen-activated protein kinase (MAPK) kinases (MEKs) [22]. It cleaves the N-termini of several intracellular MEK members viz. MEK1, MEK2, MEK3, MEK4, MEK6 and MEK7 [23, 24]. Cleavage of MEKs blocks several signal transduction pathways involved in the progression of cell cycle including the ERK (extracellular signal-regulated kinase), p38 and JNK (c-Jun N-terminal kinase) pathways [23]. These pathways are involved in cell proliferation, differentiation and survival [25]. Unlimited cell growth is a typical feature of cancerous tissues and is characterized by elevated quantities of MAPK due to its role in cell cycle progression [26]. Lethal toxin treatment resulted in partial or complete remission in a sub-cutaneous xenograft melanoma model [27]. *In vivo* treatment of fibrosarcoma, the cell dependent on mitogen activated protein kinase kinases (MEKs) revealed reduced tumor growth with reduced vascularization upon treatment with lethal toxin (LeTx) [28]. The similar results have been demonstrated by Liu et al. [29], where reduced vascularization in the tumor was observed after engineered lethal toxin treatment. MAPKs activation is the result of a cascade, which starts with the binding of ligand with the c-Met tyrosine kinase receptor (product of c-Met proto-oncogene). Upon binding, the c-Met receptor dimerizes and both the units auto-phosphorylate at tyrosine residues, which in turn creates active binding sites for proteins mediating downstream signaling [30]. This downstream signaling leads to activation of the MAPK [31–34]. Elevated level of c-Met RNA, protein and a MET transcriptional profile is linked with the mammary tumor progression and c-Met mediated MAPK cascade activation (Figure 1) [35–38]. Since LF has the inherent property to cleave MEKs, its role in anti-proliferative effect on tumors can be hypothesized. Targeting of anthrax toxin receptors (ATR) provide a strategy to inhibit tumor growth by virtue of targeting tumor vascularization due to abundance of ATR on tumor vasculature [39].

The c-Met receptor is involved in the activation of MAPK downstream signaling, growth and differentiation and known to express on surface of tumor cells [34]. Apart c-Met receptors, several other receptors are also known to participate in tumor growth especially with regard to breast cancer. The examples are nerve growth factor receptor (NGFR) [40, 41], epidermal growth factor receptor (EGFR) [42, 43], fibroblast growth factor receptors (FGFR) [44, 45] and platelet-derived growth factor receptor (PDGFR) [46]. All these are the members of tyrosine kinase receptor family and many cancer therapies against these receptors are in clinical and preclinical status [47–49]. Therefore, the effect of recombinant rLF, rPA and LeTx proteins on cultured primary mammary ductal adenocarcinoma cells and the possible interactions (*in silico*) of c-Met, NGFR, EGFR, FGFR and PDGFR with LF protein were analyzed in the present study.

## RESULTS

### *In vitro* study on primary mammary tumor cells

Residual mammary tumor biopsy tissues of mid-aged women (more than 50 years old) were obtained from Ayushman Hospital, Bhopal, M.P., India. Histopathology reports (Data not shown) of these biopsy samples identified as mammary adenocarcinoma (ductal) grade III of T2N2 stage. Cytosmears revealed loose cohesive clusters of large pleomorphic cells with very few infiltrating lymphocytes ensuring proliferative/ anti-proliferative effect of recombinant LF and PA proteins is restricted only to parenchymatous (neoplastic) cells.

### Localization of proteins and yield

**Figure 2:**
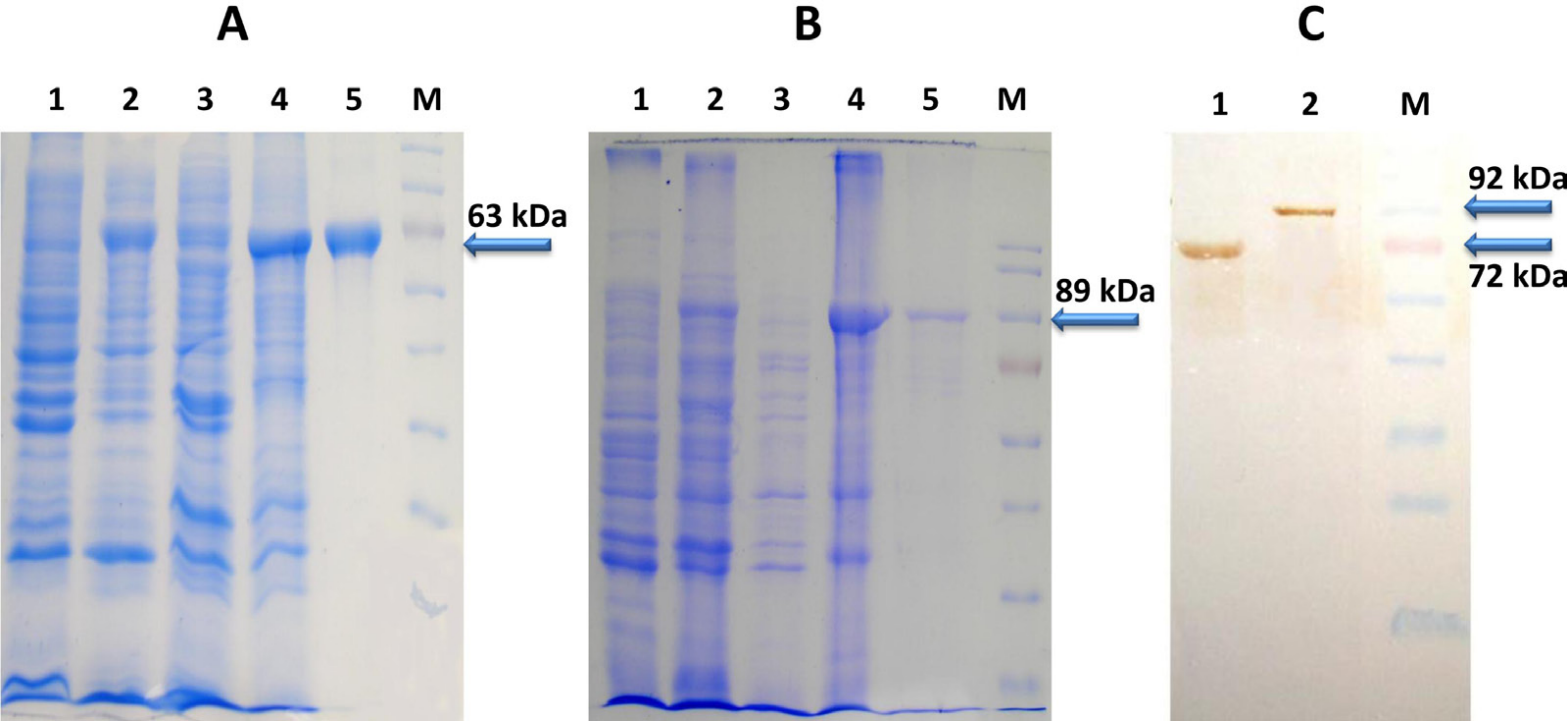
(**A**) SDS-PAGE analysis of *E. coli* expressed 6X His Tagged PA Protein (**B**) SDS-PAGE analysis of *E. coli* expressed 6X His Tagged LF Protein (Lane 1 corresponds to Rosettablue(DE3)pLysS *E.coli* cell lysate; lane 2- Total cell pellet of induced culture; Lane 3-Soluble fraction of cell lysate; Lane 4-Inclusion body fraction of cell lysate; Lane 5-Ni-NTA purified PA/ LF protein; Lane M- Molecular weight markers) (**C**) Western blot analysis of purified PA and LF proteins (~63kDa PA protein and ~85kDa LF protein) (Lane 1 corresponds to Purified PA63 protein; lane 2-Purified LF protein; Lane M- Molecular weight markers).

Both the proteins were localized into inclusion body fraction and were found specific as indicated by western blot analysis (Figure 2). The yield of rLF and rPA is 1.5 mg l^−1^ and 8 mg l^−1^ of culture respectively (rLF-85kDa; rPA-63 kDa). Both the recombinant proteins (rLF and rPA) were biologically active and possessing anti-angiogenic effect on CAM, evidenced by presence of mesodermal plexus, which failed to migrate to ectoderm. Additionally, rPA was found to cause hemorrhage in the treated CAM, indicative of its biological activity [50].

### Effect of recombinant proteins on proliferation of mammary tumor cells

**Figure 3:**
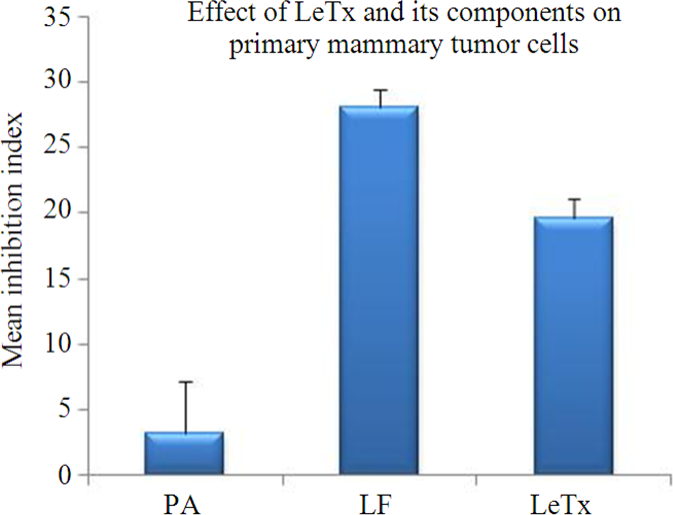
Decrease in proliferation caused by LeTx and its components was calculated as inhibition index and the values are shown as the mean ± SD for a minimum of three independent replicates.

The results of the present study showed that both rLF and LeTx significantly (*p* < 0.01) reduced the proliferation of mammary tumor cells with mean inhibition indices of 28.0 ± 1.37 per cent and 19.6 ± 1.47 per cent respectively, however treatment with rPA alone had no statistically significant anti-proliferative effect as indicated by low mean inhibition index of 3.4 ± 3.87 percent (Figure 3). Since the LF is a metalloprotease and having capacity to cleave MAPK, it may be effective against several tumors, where cell cycle progression is largely dependent on MAPK signaling.

Here, it is noteworthy that rLF alone has caused more inhibition than LeTx, showing biological activity of the proteins produced as well as LF exhibited enzymatic function independent of PA, which have key role in trafficking of LF inside the cell. So presumably, apart from PA, another receptor might exist for LF, which led to the further investigation through *in silico* analysis.

### *In silico* protein docking analysis reveals interaction between LF and c-Met receptors

**Figure 4:**
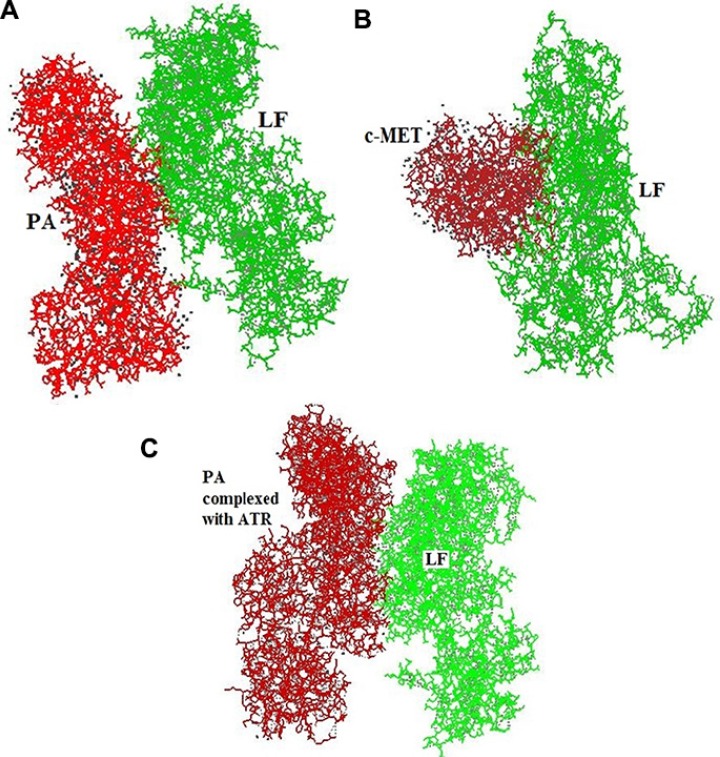
*HEX-8* software generated docking model for (**A**) LF-PA interactions (**B**) LF-c-Met interactions (**C**) LF-PA interactions after complexing with ATR.

The results of *in silico* protein docking analysis of different receptors with LF have been given in Table 1. In the condition of anthrax toxin receptor (ATR) bound PA, the PA-LF interaction had 22 H bonds and a free energy value of −402.6, indicating higher number of H bonds but higher free energy. The software generated docking models for LF-PA, LF-PA-ATR and LF-c-Met interactions has been depicted in Figure 4A to 4C. Among the other receptors, NGFR and HER-1 showed good interactions with LF in terms of higher number of H-bonds and higher free energy values (Table 1). The present *in silico* analysis using HEX software, revealed a stronger interaction of c-Met and LF, suggesting c-Met as alternative receptor for LF traffic inside the cell or for modulating the signaling cascade upon binding.

**Table 1:** Results of *in silico* protein docking of receptors involved in cell proliferation and mammary tumor with LF using *HEX*-8 software

S. No.	Name of receptor docked with LF protein (1JKY) (*B. anthracis*)	No. of hydrogen bonds	Free energy (e-total)
1	Hepatocyte growth factor receptor (c-Met receptor) (3DKC)	19	−773.96
2	Nerve growth factor receptor TrkA (1HE7)	15	−561.83
3	Human Epidermal Growth Factor HER1 (2ITX)	13	−765.30
4	Human Epidermal Growth Factor HER2 (3PP0)	11	−388.29
5	Human Epidermal Growth Factor HER3 (1M6B)	20	−145.42
6	Human Epidermal Growth Factor HER4 (3BCE)	4	−260.00
7	Human Platelet-Derived Growth Factor (1PDG)	7	−577.35
8	Fibroblast Growth Factor Receptor (1FGK)	2	−412.82
9	Protective antigen (*B. anthracis*) (4H2A)	12	−420.48
10	Protective antigen bound to Anthrax toxin receptor (1T6B)	22	−402.6

**Figure 5:**
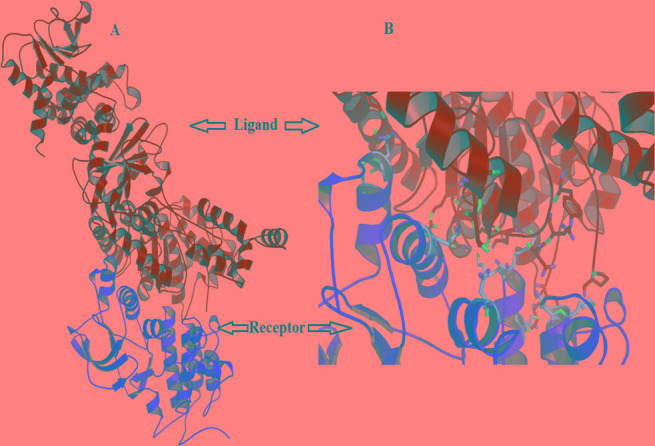
(**A**) The site of interactions of c-Met receptor with the LF using ClusPro (**B**) enlarged view of the same.

The *in silico* protein docking analysis revealed the presence of a stronger interaction between LF and c-Met receptor (H bond = 19; and Free energy = −773.96) in comparison to that of between LF and PA (H bond = 12 and Free energy = −420.48). The data indicates that LF have stronger affiliation with c-Met in contrast to its natural counterpart PA. Since, the number of H bonds and minimum free energy are the indicator of stronger interaction and higher affinity. The portion of c-Met receptor between amino acid residues no. 1061 to 1335 interact with the amino acid residues no. 409 to 704 of LF (Table 2, Figure 5).

**Table 2:** The suitable model for the site of interaction; between the amino-acid residues of c-Met receptor and LF using ClusPro

Residue no. of c-Met receptor	Residue no. of LF
E1061	K552
E1064	Q560
Q1123	R409
D1133	N626, R628
K1193	Q704, N703
K1198	E662, N626, G625
K1199	E662, H645
D1231	K410
K1259	E648, Y650
T1262	D647
K1263	P16
S1331	Q704
S1335	Q704

## DISCUSSION

Mammary cancer is one of the most common cancers present among the females and accounts for approximately 25% deaths due to cancer. Several FDA approved drugs are being given as part of chemotherapy for treating breast cancer including Epirubicin, Doxorubicin, Daunorubicin and Cyclophosphamide, acting by damaging cancer cells and killing them; Everolimus, acting by inhibiting mTOR kinase; Pamidronate, acting by limiting the action of osteoclastic cells and thereby preventing metastasis in bone from mammary tissues, Anastrozole and Raloxifene by reducing the relapse of hormone-receptor-positive breast cancer; Bevacizumab, the only treatment act by targeting angiogenesis in breast cancer; Paraplatin, the only platinum base therapy, acting through damaging genetic material etc. However, these are all associated with some common problems including enhanced risk of stroke, headache, nausea, vomiting, abdominal pain, joint pain, muscle pain, diarrhea or constipation [51, 52]. Trastuzumab, a humanized mAb targeting the HER2 receptor, displays a good overall survival of patient [53] however; pose threat of cardiac dysfunction [54]. Another approach to circumvent the mammary tumor cell growth is to target MAPK, a key enzyme in the Ras-Raf-MEK-Extracellular signal Regulated Kinase (ERK) kinase pathway, which is constitutively active in tumors including mammary tumor [55, 56]. LeTx, a binary toxin produced by *B. anthracis* is catalytically a potent inhibitor of the MAPK pathway. It binds and internalizes within most of the tissues but is toxic only to cells dependent on MAPK signaling for survival. Considering the fact that mammary tumor cells over-express MAPK, which is an enzymatic substrate of lethal factor; hence, the inhibition in mammary tumor cells proliferation by LeTx can be hypothesized. Therefore, the effect of LeTx and its components (rLF, rPA) on proliferation of primary mammary tumor cells was evaluated *in vitro*.

Cell lines are widely accepted models for evaluation for antitumor therapeutic drugs, for retaining many genetic, epigenetic and gene expression features [57], but are more complex than tumor itself due to extensive chromosomal rearrangements, oncogene mutations, and multiple sites of allelic loss, gene amplification and probable change in some cellular pathways [58]. The difference in degree of aneuploidy and steroid receptor status between breast tumor and breast cancer cell lines [59–61] makes cell lines non-representative of the most common diverse type of tumors.

Different concentrations of PA and LF, ranging from 100 ng to 1 μg [62–66], have been used previously to see their cytotoxic effect on LeTx sensitive mouse macrophage like cell lines RAW264.7 and J774A and at this concentrations cytotoxicity was observed. Though cytotoxic dose of LeTx on mammary tumor cells is not known, a dose of 50 ng of lethal factor, nontoxic even to LeTx sensitive cells, was used, to be assured that the effect of the recombinant protein on primary mammary cells is attributed to enzymatic or other cellular function and not due to cytotoxicity. The LF to PA ratio ranging from 1:3 to 1:5 has been demonstrated to have maximum anti-proliferative activity on melanoma tumor [67]. This information support the explanation of dosage of rLF to rPA in LeTx used in the present study (1:3 with 50 and 150 ng of each antigen respectively).

Inhibition in proliferation of mammary tumor cells by LF and LeTx in this study (inhibition indices 28 and 19%) demonstrates the potential use of LF and LeTx as therapeutic agent against tumors. Tumor endothelium marker-8 (TEM-8) and capillary morphogenesis protein-2 (CMG2) are the two types of ATR present on the surface of host cells. TEM8 is selectively upregulated in endothelial cells during blood vessel formation and in endothelial cells of neoplastic tissue; therefore toxicity of lethal toxin of *B. anthracis* may be targeted specifically to growing tumor vasculature [39]. This data further may be exploited in targeting solid tumors. Further studies are required to test their *in vivo* efficacy. Current literature on anthrax toxin activities states that LF and EF as individual proteins are inactive and they become functional only after binding to PA as binary toxin (PA + LF = LeTx; PA + EF = EdTx) [68, 69]. Protective antigen has been long considered as trafficking moiety facilitating entry of LF and EF into the cells through clathrin, actin and unconventional receptor mediated endocytosis [39, 70]. In a previous study, intracellular expression of LF in human lung adenocarcinoma cell line was found to cause cytotoxic effect [71]. Contrary to the existing knowledge of PA dependent trafficking of LF, the higher inhibition index observed in the study for rLF alone as compared to that of LeTx is indicative of enzymatic activity of LF alone without the need of PA.

On the basis of above results, PA independent receptor mediated endocytosis or modulation of signal transduction cascade via binding to other unknown moieties can be hypothesized. To elucidate the possible interaction of LF with other cellular receptors i.e. c-Met, NGF, EGFR, FGF and PDGF, predominantly overexpressing on mammary tumor cells were checked *in silico*. NGF is reported to participate in neuronal cell survival and differentiation and there are growing evidences of role of NGF as major stimulator of breast cancer cell growth. The action of NGF is mediated by TrkA (tyrosine kinase receptor family) and p75NT (tumor necrosis factor receptor family) [40]. TrkA also known as high affinity nerve growth factor receptor and is major receptor [72]. Upon binding with ligand, it undergoes autophosphorylation and cascade of MAPK phosphorylation starts. p75NTR is a minor receptor which upon interacting with TrkA receptor, form high-affinity binding sites for NGF [73, 74]. Being major receptor, TrkA receptor interaction was taken into account. It showed 15 hydrogen bonds and -561.83 of free energy. The second receptor, epidermal growth factor receptor (EGFR) is observed to be over expressed in all subtypes of breast cancer. The members of the epidermal growth factor receptor are EGFR (HER1), HER2 (also known as ErbB2), HER3 (also known as ErbB3), and HER4 (also known as ErbB4) [42]. Upon growth-factor binding, EGFR family members homo- or hetero-dimerize and activate their cytoplasmic tyrosine kinase domains to initiate intracellular signaling [75, 76]. Upon subjecting all EGFR interaction with LF, maximum interaction was shown by HER1 with 13 hydrogen bonds and −765.30 of free energy. Though HER3 exhibited more numbers of hydrogen bonds (20), but its free energy was more. HER2, which is over-expressed in 20% to 25% of breast cancers and is the well established therapeutic target in breast cancer [77] showed only 11 hydrogen bonds with -388.29 energy. HER4 receptor showed even less degree of interaction. The PDGF, are tyrosine kinase receptors, function in controlling development of mesenchymal cells, such as pericytes, fibroblasts and vascular smooth muscle cells [78]. In breast cancer, correlation of PDGF β-receptor expression is found [79]. The PDGF β-receptor also showed less degree of interaction (H bond = 7 with free energy = −577.35). FGFR family comprises four members, FGFR-1, -2, -3, and -4 [80]. The activation of signalling cascade involve binding of ligand to the extracellular domain of receptor and phosphorylation of the cytoplasmic tail of the receptor followed by activation of rat sarcoma mitogen-activated protein kinase (RAS–MAPK) pathway. The several studies have identified amplifications of FGFR1 in breast cancer [81, 82] and hence included in the study, but there are studies disapproving its role in cancer progression also [83]. In our study, poor interaction of FGFR with LF is observed *in silico*. The c-Met receptor, a product of proto-oncogene c-*met* [84], is a known tyrosine kinase receptor involved in many signaling pathways associated with growth, differentiation, motility, migration and invasion. Up regulation of c-Met receptor and MAPKs activation leading to cell proliferation has been reported in progressive mammary tumors [36]. Apart mammary cancer [85], cMET has been expressed in several cancers including advanced esophageal squamous cell carcinoma [86], lung cancer [87], Renal Cancer [88], Malignant skin cancer [89], pancreatic cancer [90] etc. Recently, inhibition of cMET has been demonstrated to display therapeutic effects in ovarian clear cell carcinoma (OCCC). Use of cMET inhibitors, like SU11274 or crizotinib, induce apoptosis and reduce proliferation of OCCC cells. Other inhibitors include other cMET targeting therapies for treating cancers including Monoclonal antibodies including Rilotumumab and Onartuzumab; Small molecule c-Met tyrosine kinase inhibitors (TKIs) including Tivantinib (ARQ197), AMG337 or Foretinib and c-Met targeting antibody ABT-700 [91, 92].

*In silico* docking analysis was performed to understand the possible interactions between LF and c-Met and compared to LF-PA as well as LF-PA-ATR interactions and it revealed a strong interaction between LF and c-Met receptor, as evident by presence of higher number of H bonds [19] and lower free energy (−773.96) in comparison to its natural trafficking molecule PA. The higher number of H bonds [22] observed between LF and PA bound to ATR may be due to the conformational change in PA induced by PA-ATR interaction. Although the number of H bonds in the LF-c-Met interaction is less than that in the LF-PA-ATR interaction, the presence of lower free energy exhibited a thermodynamically more stable interaction due to the non-availability of energy to collide and react with other molecules. The results of this analysis suggests that out of 5 receptors envisaged in the study, LF binds with c-Met receptor strongly and possibly compete with other ligands which are involved in MAPK mediated cell proliferation pathways, leading to an inhibitory effect on tumor growth. Though, involvement of NGF and HER1 receptors can't be denied. Hence, we suggest c-Met receptor as one of the major possible molecule involved in the alternative strategy adapted by LF to perform its action in a PA independent manner either by modulating cellular signaling cascades or through LF internalization. Further studies are required in this direction.

## MATERIALS AND METHODS

### Recombinant *B. anthracis* proteins

The LF and PA genes were amplified using bacterial plasmid DNA as template. Primer F- GCTAGCATTACTTTGAGTGGTCCCGTCTTT; Primer R-TCTAGAATGGCTGGTCCCGTTATT and Primer F- AGTGCTCTCGAGACGGTTCCAGACCGTGAC Primer R–AAATCACGATCGATTACCTTACCTATCTC were used for amplification of LF and PA genes respectively. After a cloning step in pGEM-T easy vector (Promega, Madison, WI, USA) for sequencing, these genes were subcloned into pQE-31 (Qiagen, India) for LF gene and pET28(c)^+^ for PA63 (Novagen, Billerica, MA, USA) respectively. The constructs were transformed into their respective expression host SG13009 and Rosetta blue (DE3) codon plus *E. coli* cells. Recombinant protein expression was induced by adding 1mM IPTG and the culture was induced for 4 hours. The cells were harvested by centrifugation and recombinant proteins were purified using Bug buster protein extraction reagent (Novagen). Soluble and inclusion body fraction was collected separately and run on 12.5% SDS polyacrylamide gel. The protein was characterized by western blot analysis using polyclonal serum raised in monkey against attenuated live anthrax spore vaccine. The recombinant proteins were purified using His Bind purification kit (Novagen) and subjected to refolding by protein refolding kit (Novagen) as per manufacturer protocol [93]. The protein was quantified using Qubit protein assay kit (Life Technologies, California, US). Biological activity of both the proteins was determined in chorioallantoic membrane (CAM) of embryonated chicken eggs for their effect on process of vascularization.

### *In vitro* study on primary mammary tumor cells

Residual mammary tumor biopsy tissues were provided by Dr. Sunita Yadav, MS, General and Cancer surgeon, Ayushman Hospital, Bhopal, M.P., India. The tissue were dissected and trypsinized (0.01%) in RPMI medium. Cells were resuspended in RPMI medium supplemented with 10% FBS and seeded in a 96 well plate at a concentration of 5 × 10^3^ cells per well in 100 μl volume. Per microscopic field 3-4 infiltrating lymphocytes were present. Four test groups were prepared, each comprising triplicate wells. 100 μl each of rPA (150 ng/well), in first set; rLF (50 ng/well) in second set; and LeTx [mixture of rPA (150 ng) + rLF (50 ng)] in third set of experiment were added in triplicate in 96 well plate (Nunc). For control group, 100 μl RPMI media was added.

The plate was incubated at 37°C in CO_2_ (5%) incubator for 72 h. Then 100 μl of cell suspension was taken out and mixed with 50 μl of MTT solution (5 mg ml^−1^). The plate was incubated at 37°C for additional 4 h. Resulting formazon crystals were dissolved in 100 μl of DMSO by vigorous pipetting and incubated at 37°C for 15 min. Optical density (OD) was measured at 492 nm in a multi-well ELISA plate reader (Tecan, Japan). Inhibition index was calculated from the OD values using the following formula:

$$\text{Inhibition index}=\frac{\text{OD of control group−OD of treated group}}{\text{OD of control group}}$$

The significance of difference between test groups was analyzed by student's *t-test* and *p-value* less than 0.01 was considered as significantly different.

### *In silico* docking analysis

In order to compare and understand the interaction of LF (PDB: 1JKY) with PA (pH7.5; pdb-4H2A), PA bound to Anthrax Toxin Receptor (ATR) (PDB: 1T6B), c-Met (PDB: 3DKC) and other related group of receptors (NGFR, EGFR, FGFR and PDGFR); *in silico* analysis was carried out using *HEX-*8 protein docking software (http://hex.loria.fr). Number of hydrogen bonds involved in the interactions between the docked proteins and the total free energy for each interaction were compared. Presence of higher number of H bonds and lower free energy indicated a stronger interaction between the proteins. The interaction between receptors and ligand was also confirmed using software ClusPro.

## CONCLUSIONS

Results of the present study demonstrated the potential of rLF and LeTx for use as alternative therapeutics against mammary ductal carcinoma. Since mammary tumors are highly heterogeneous, the effect of LF and LeTx on other mammary tumor categories, need to be evaluated further. As indicated by the higher inhibition index observed for rLF alone as compared to that of LeTx, possible existence of PA independent modes of action of LF such as receptor mediated PA independent endocytosis or modulation of signal transduction cascade via other unknown interactions was hypothesized. *In silico* docking analysis also revealed the plausible existence of c-Met as an alternative receptor for LF to mediate and modulate PA independent signal transduction.
